# Updates on food and feed mycotoxin contamination and safety in Africa with special reference to Nigeria

**DOI:** 10.1080/21501203.2021.1941371

**Published:** 2021-06-20

**Authors:** Francis Imade, Edgar Mugizi Ankwasa, Hairong Geng, Sana Ullah, Tanvir Ahmad, Gang Wang, Chenxi Zhang, Oyeyemi Dada, Fuguo Xing, Yongquan Zheng, Yang Liu

**Affiliations:** a Institute of Food Science and Technology, Chinese Academy of Agricultural Sciences /Key Laboratory of Agro–Products Quality and Safety Control in Storage and Transport Process, Ministry of Agriculture and Rural Affairs, Beijing, China; b School of Food Science and Engineering, Foshan University/South China Food Safety Research Center, Guangdong, China; c State Key Laboratory for Biology Pests, Institute of Plant Protection, Chinense Academy of Agricultural Sciences, Beljing, China; d Botany Department, Faculty of Life Sciences, Ambrose Alli University, Ekpoma, Edo State, Edo State Nigeria; e Department of Crop Protection and Environmental Biology, University of Ibadan, Nigeria

**Keywords:** Mycotoxin, contamination, international trade, agricultural commodities

## Abstract

Mycotoxin contamination of food and feed is a major concern in sub-Sahara African countries, particularly Nigeria. It represents a significant limit to health of human, livestock as well as the international trade. Aflatoxins, fumonisins, ochratoxin, zearalenone, deoxynivalenol and beauvericin are the major mycotoxins recognised in the aetiology of food safety challenges that precipitated countless number of diseases. In Nigeria, aflatoxins and fumonisin found in nearly all crops are the most common mycotoxins of economic and health importance such as sorghum, maize and groundnuts. Thus, consumption of food contaminated with mycotoxins are inevitable, hence the need for adequate regulation is necessary in these frontier economies as done in many developed economies to ensure food safety for human and animals. In low and middle-income countries, especially Nigeria, there is lack of awareness and sufficient information on the risk associated with consequent of mycotoxin contamination on wellbeing of human, animals health and the economy. It is based on the foregoing that this paper summarized the status of mycotoxin present in Nigerian food and feeds relative to the global regulatory standards. This aimed at preventing consuming mycotoxin contaminated food stuff while confronting its associated challenges. Suggestions on some possible control strategies to mitigate vending mycotoxin food and feeds were made.

## Introduction

Mycotoxins are toxic secondary fungal metabolites frequently found as contaminants in food and feed with attendant negative effects on humans and animal’s health when ingested. They are fungal secondary metabolites that may develop in almost any food or feedstuff during the growing season, at harvest time, or during processing or storage, depending on the environment and method of handling (Abass et al. 2017). It can as well develop in bottled water as reported by Mata et al. (2015). The occurrence of mycotoxin in food and feed chains is ubiquitous, affecting both human and animal health as well as economically affecting developing and developed countries via exposure to variable concentrations.

Despite the differences in factors that influences mycotoxin contamination, exposure effect is completely global (Ladeira et al. 2017). Exposure either for short or long term can lead to broad toxic effect due to toxicity of mycotoxins diversity (Abdallah et al. 2020) or mainly through direct consumption or metabolite carried over in animal products.

Recently, contamination of food and feed by mycotoxin is a global concern day by day and have caused serious out breaks worldwide affecting human and animal health, as well as losses in nations economy (Cinar and Onbaşı 2019). It is estimated that 25% of world’s food crop is contaminated by fungi-producing mycotoxins, causing huge losses in billions of dollars in domestic and international trade involving agricultural products (Atanda et al. 2013; Pankaj et al. 2018; Cinar and Onbaşı 2019). In 2017, it was reported that high level of mycotoxins content in food are the cause for the loss of 617 billion naira which Nigeria could have realised from non-oil export in nine years if the level of contamination were below safe limit (https://businessday.ng/).

According to Peraica et al. (2014) young ones including animals and children are highly sensitive and show reactions to effect of mycotoxins than adults due to their lower body mass, higher metabolic rate, and underdeveloped organs and detoxification mechanisms. Despite the bulk of research on assessment of mycotoxins contaminated food and its exposure, the public health impact is largely ignored in Nigeria at the expenses of rising incidence that are linked to cancers, immune system defects, growth retardations, liver diseases and death due to short term or long term exposure (Adjovi et al. 2015; Muhammad et al. 2019).

Contamination of food and feed by mycotoxin has been reported to be both direct and indirect (JPF and Macdonald 1997; Tola and Kebede 2016). Direct contamination explains a situation when food and feed commodities are tainted with mycotoxin-producing fungus. A condition whereby toxigenic fungus is no longer in the contaminated food/ingredient used in the production of food but the toxin released are present due to their resistant to processing procedures is an indirect contamination (Alshannaq and Yu 2017; Sokefun et al. 2018). This has led to a situation where the quality of such commodities were compromised causing negative effects on international trade (Dikhoba et al. 2019). This also poses a huge threat to human and animal health as the food crops are rejected for not meeting international trade standards are left for the average or low-income earners to consume. This is a common occurrence in developing countries like Nigeria, Ghana, Republic of Benin, Lesotho, Ethiopia, Kenya and many others.

As a consequent of deleterious effects of mycotoxin on human and animal health, several countries have implemented several regulations which prescribe the limits/allowable concentration of mycotoxin in several food and feed commodities (Mu et al. 2017). Researches focused on major agricultural and human health related mycotoxins such as fumonisins, zearalones, deoxynivalenol, T-2, aflatoxins and ochratoxins which mostly infect maize, wheat and rice (Alshannaq and Yu 2017; Onyedum et al. 2020) among other emerging mycotoxins such as enniatins, beauvericin, moniliformin, fusaproliferin, fusaric acid, culmorin, butenolide, sterigmatocystin, emodin, mycophenolic acid, alternariol, alternariol monomethyl ether, and tenuazonic acid (Gruber-Dorninger et al. 2017). The negative effect of mycotoxins has affected the world economically by hampering international trade due to rejection of mycotoxin contaminated food and feed that are above the regulatory limit (Abdallah et al. 2020). Food safety and security has been a major issue in many frontier countries due to unregulated nature of food and feed products. Abass et al. (2017) reported that that dried cassava products are potential sources of mycotoxin contamination in human diet in Nigeria. The production of various indigenous African food such as *injera, banku, amasi, fufu, garri* produced in home under spontaneous conditions with little or no hygienic control could also contribute to high mycotoxin contamination (Adekoya et al. 2017a). It is supposed that multiple mycotoxins in food and feed are direct consequence of poor and none compliant with quality control measures such as regulation of moisture content in grains (Adekoya et al. 2018).

Olufunmilayo and Oyefolu (2010) carried out an assessment of mycotoxin exposure on poultry meat consumption in major markets across Oyo state, Nigeria and isolated a lot of fungi genera belonging to *Aspergillus* spp., *Alternaria* spp., *Fusarium* spp., *Cladosporium* spp., *Penicillium* spp., *Neurospora* spp., *Rhizopus* spp. and yeast. Three of these genera, *Aspergillus, Fusarium* and *Penicillium,* are potential mycotoxin-producing fungi. The heavy fungi load was attributed to poor housing condition.

It was found that aflatoxin group members were present at an insignificant concentration lower than the African permissible limit of 5 and 20 mg/kg for AFB1 and total AFT, respectively. Though, low concentration was recorded in the samples at the time of the study, but urgent attention is needed to address the causes of the contamination so that accumulation would not attain the dangerous dimension that could result in human health issues.

It is also important to note that, in spite of existence of mycotoxin regulations in most countries, the effect of multiple occurrence of mycotoxins is not usually considered when establishing these limits. Adekoya et al. (2018) isolated nine mycotoxins – deoxynivalenol, DON; neosolaniol, NEO; fusarenon-X, FUS-X; enniatin B, ENN B; alternariol, AOH; fumonisins B1 and B2, alternariol monomethyl ether, AME and Sterigmatocystin STERIG – co-occurring in South African maize-based opaque beer. This underscores the fact that the occurrence of these mycotoxins could confer some health risks hence the need for further studies and recommendations that addresses co-occurrence of mycotoxin is expedient. Information is scanty on losses associated with mycotoxin contamination in tropical and sub-tropical countries, incidence of its occurrence in food products especially agricultural commodities is glaring. Hence, the non-existence of regulatory limits to protect consumers from severe health risk linked to mycotoxins (Chilaka et al. 2018b). Contamination of agricultural products by fungi infection and the production of mycotoxins have also been linked to the influenced by climate change (Van Der Fels-Klerx et al. 2016). The climatic conditions of Nigeria are characteristically tropical with high temperature and humidity which favours proliferation of fungi that releases toxic secondary metabolites leading to unprecedented food and feed contamination.

According to Chilaka et al. (2019), several studies have elucidated the activities of mycotoxin such as *Fusarium* during food processing. However this is contrary to situation in Nigeria where very little information is known particularly with respect to traditional food preparation and the quantity of mycotoxin involved in food consumed by humans. This paper therefore summarises the current status of quantifiable mycotoxin present in Nigerian foods in comparison to the regulatory standard set to prevent human and animals from consuming contaminated food and to provide some possible control strategies that could be adopted.

## Mycotoxins contamination of food and feeds in Nigeria

**Figure 1.**
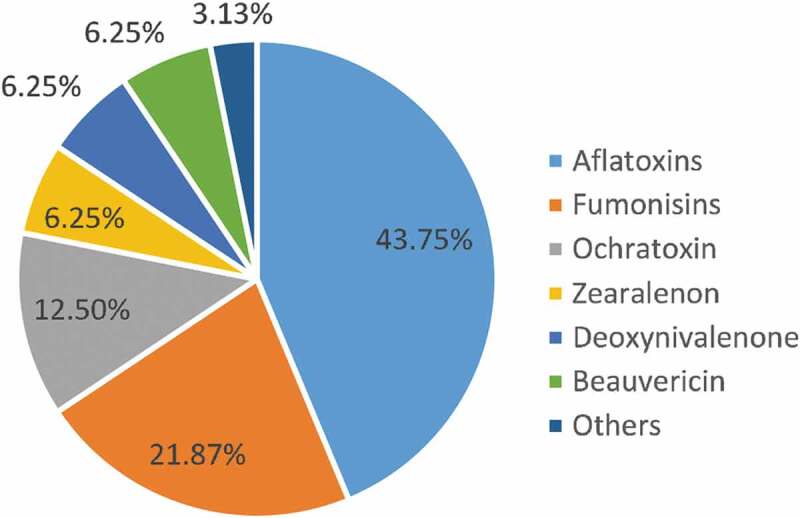
Percentage of contamination rate of concern mycotoxin in sub-Saharan Africa. Modification of Darwish et al. (2014)

In sub-Saharan Africa (SSA), the prevalent mycotoxins of concerned affecting the health of human, animals and economy are aflatoxins (43.75%) followed by fumonisins (FUM, 21.87%), ochratoxin (12.5%), zearalenone (ZEN, 9.38%), deoxynivalenol (DON, 6.25%), beauvericin (BEA, 6.25%) (Darwish et al. 2014), while others constitute 3.13% (Figure 1). Globally in 2019, data from January to December showed the most prevalent mycotoxins were FUM (70%) and DON (68%) (BIOMIN World mycotoxin survey 2020). A 10-year survey by Gruber-Dorninger et al. 2019 in SSA, reviewed an increased shift in prevalence in Fumonisins (72.6%); ZEN (52.2%) and DON (49.5%) amidst other emerging mycotoxins (Ladeira et al. 2017; Chilaka et al. 2018b; Ojochenemi et al. 2019; Ikeagwulonu et al. 2020; Kebede et al. 2020). Table 1 shows an update of mycotoxin contamination of some food and feed commodities in different locations across Nigeria.

**Table 1.** Update on mycotoxins contamination of food and feed commodities in different locations across Nigeria

Food and feed commodities	Location	Mycotoxins	Concentrations (range/mean)	References
Maize	Southeastern Nigeria	AFT	0.07–109.78 μg/kg	Egbuta et al. (2015)
	Southeastern Nigeria	FB1	10.0–3644 μg/kg	Egbuta et al. (2015)
	Niger state	OTA	0–139.2 μg/kg	Makun et al. 2013)
	Anambra state	ZEN	170 μg/kg	Oyeka et al. (2019)
	Southeastern Nigeria	DON	0.1–0.7 μg/kg	Egbuta et al. (2015)
	Kogi state	DON	1.34–9.25 μg/kg	Akoma et al. (2019)
	Anambra state	DON	58.2–1890 µg/ kg	Oyeka et al. 2019)
	Southeastern Nigeria	ZEN	1.8–652.3 μg/kg	Egbuta et al. (2015)
	Calabar. Cross River state	ZEN	54.79–54.84 μg/kg	Neji et al. (2018)
	Southeastern Nigeria	OTA	0.6–79.0 μg/kg	Egbuta et al. (2015)
	Agro-ecological zones	T-2	0–29 μg/kg/11.9 µg/kg	Afolabi et al.(2013)
	Calabar. Cross River state	Aflatoxin B1	1.73 −1.78 μg/kg	Neji et al. (2018)
	Calabar. Cross River state	Ochratoxin A	0.50– 0.54 μg/kg	Neji et al. (2018)
	Calabar. Cross River state	Fumonisin B1+ B2	139.45– 139.47 μg/kg	Neji et al. (2018)
Ginger	Lagos state	AFT	0.11–9.52 μg/kg	Lippolis et al. (2017)
Cowpea: white and brown	Lagos, Ogun and Oyo	AFT	209 µg /kg White 88 µg /kg brown	Afolabi et al. (2020)
Cowpea	Ibadan, Oyo state	AFB1	1.5 x 10–2 µg/g,	Ogungbemile et al. (2020)
	Ibadan, Oyo state	AFB2	0.80 x 10– 2 µg/g,	Ogungbemile et al. (2020)
	Ibadan, Oyo state	AFG1	0.60 x 10–2 µg/g,	Ogungbemile et al. (2020)
	Ibadan, Oyo state	AFG1	1.0 x 10– 2 µg/g	Ogungbemile et al. (2020)
Millet	North–Central Nigeria	AFT	4.80–45.60 μg/kg	Onyedum et al. (2020)
	Calabar. Cross River state	ZEN	55.52–55.60 μg/kg	Neji et al. (2018)
	North–Central Nigeria	OTA	1.80–6.20 μg/kg	Onyedum et al. (2020)
	North–Central Nigeria	Fum	10–8400 μg/kg	Onyedum et al. (2020)
Millet grain	Niger state	AFT	1.05–14.96 μg/kg	Apeh et al. (2016)
Millet dough	Niger state	AFT	0.81–3.78 μg/kg	Apeh et al. (2016)
Sorghum	North–Central Nigeria	AFT	4.80–42.60 μg/kg	Onyedum et al. (2020)
	North–Central Nigeria	Fum	50–2510 μg/kg	Onyedum et al. (2020)
	North–Central Nigeria	OTA	1.40–5.60 μg/kg	Onyedum et al. (2020)
Corn	North–Central Nigeria	Fum	110–5110 μg/kg	Onyedum et al. (2020)
Yam flour	North–Central Nigeria	AFT	5.0–39.45 μg/kg	Onyedum et al. (2020)
	North–Central Nigeria	OTA	1.20–8.20 μg/kg	Onyedum et al. (2020)
	North–Central Nigeria	Fum	10–7200 μg/kg	Onyedum et al. (2020)
Garri	North–Central Nigeria	AFT	2.60–55.40 μg/kg	Onyedum et al. (2020)
	North–Central Nigeria	Fum	10–1390 μg/kg	Onyedum et al. (2020)
	Anambra state	T-2	9–22 μg/kg	Chilaka et al. (2018a)
	North–Central Nigeria	OTA	1.30–170.1 μg/kg	Onyedum et al. (2020)
Unprocessed flour/grain	Northern Nigeria	FB1	2.7–10,904 μg/kg	Ezekiel et al., (2019b)
Rice	Southwestern Nigeria	AFT	0.01–6.50 µg/kg	Egbuta et al. (2015)
	North Central Nigeria	AFT	2.10–248.20 μg/kg	Onyedum et al. (2020)
	Southwestern Nigeria	ZEN	0.7–570.6 μg/kg	Egbuta et al. (2015)
	Southwestern Nigeria	OTA	0.7–180.9 μg/kg	Egbuta et al. (2015)
	North–Central Nigeria	OTA	1.20–16.9 μg/kg	Onyedum et al. (2020)
	Southwestern Nigeria	DON	0.1–0.7 μg/kg	Egbuta et al. (2015)
	Southwestern Nigeria	FB1	0.9–59.6 μg/kg	Egbuta et al. (2015)
	North– Central Nigeria	Fum	10–1200 μg/kg	Onyedum et al. (2020)
	Calabar.Cross river state	ZEN	54.24–54.27 μg/kg	Neji et al. (2018)
Rice: stored, field and market samples	Niger state	T-2	NA	Makun et al. (2011)
Fin fish	Lagos state	AFT	1.05–10.00 μg/kg	Olajuyigbe et al. (2014)
Shell fish	Lagos state	AFT	4.23–5.90 μg/kg	Olajuyigbe et al. (2014)
Fermented African oil bean seed	Southwest Nigeria	AFB1	3–36. μg/kg	Adekoya et al. 2017b)
African locust beans	Kaduna and Anambra state	T-2	492 μg/kg	Chilaka et al. (2018b)
	Northern Nigeria	FB1	564 μg/kg	Chilaka et al. (2018c)
	Northwest Nigeria	T-2	28–31 μg/kg	Adekoya et al. 2017b)
Poultry feed and feed ingredient	Adamawa, Benue, Borno, Delta, Kaduna, Katsina, Kebbi, Lagos, Niger, Oyo, Rivers, and Taraba	FB1	37–3760 μg/kg	Akinmusire et al. (2019)
	Adamawa, Benue, Borno, Delta, Kaduna, Katsina, Kebbi, Lagos, Niger, Oyo, Rivers, and Taraba state	AFB1	0.5–760 μg/kg	Akinmusire et al. (2019)
Diary feeds	Kaduna state	AFB1	0.5– 24.8 μg/kg	Omeiza et al. (2018)
Fermented melon	Southwest Nigeria	AFB1	1.05–10.00 μg/kg	Adekoya et al. 2017b)
Bread	Kogi, Lagos, Ibadan Ogbomosho, PortHarcout	FB1	10–220 μg/kg	Ojochenemi et al. (2019)
Unprocessed flour/grain	Northern Nigeria	FB1	2.7–10,904	Ezekiel et al. (2019b)
Cashew nut	Abia, Kogi, Oyo, Taraba, Enugu, Anambra, Delta Benue and Lagos state	ZEN	788 μg/kg	Adetunji et al. (2019)
Wheat	Calabar. Cross River state	ZEN	55.39–55.42 μg/kg	Neji et al. (2018)
	Calabar. Cross River state	Aflatoxin B1	1.40– 1.42 μg/kg	Neji et al. (2018)
	Calabar. Cross River state	Ochratoxin A	0.42– 0.44 μg/kg	Neji et al. (2018)
	Calabar. Cross River state	Fumonisin B1+ B2	131.10– 131.12 μg/kg	Neji et al. (2018)
Egusi	Oyo, Niger Adamawa and Kebbi state	AFB1/AFG1	14.1–109.5 ng/g	Obani et al. (2019)
Melon	Benue and Nasarawa states	OTA OTB	112 μg/kg 94 μg/kg	Esan et al. (2020)
Burukutu	Nigeria *Not indicated	DON	61–255 ug/L	Chilaka et al. (2018b)
Wheat	Ogun, Oyo and Lagos state	DON	119–2560 μg/kg	Egbontan et al. (2017)
Sorghum	Minna and Bida,	AFT	0.96–21.74 μg/kg	Apeh et al. (2016)
Burukutu	Niger state	AFT	1.27–8.82 μg/kg	Apeh et al. (2016)
Pito	Niger state	AFT	0.69–2.00 μg/kg	Apeh et al. (2016)
Sesame	Jigawa Nassarawa and Niger state	AFT	0.79–60.05 μg/kg	Apeh et al. (2016)
Melon	Benue and Nasarawa states	OTA OTB	112 μg/kg 94 μg/kg	Esan et al. (2020)

FUM: fumonisins, OTA ochratoxin A, OTB: ochratoxin B, AFT: total aflatoxin; AFB1: aflatoxin B1; AFG1: aflatoxin G1; DON: deoxynivalenol; ZEN: zearalenone.

* Region/state/city not indicated.

### Aflatoxin contamination of food and feed

Owing to fungal infection, fungi-producing aflatoxins have been reported in agricultural produce, such as groundnuts, millet, sesame seeds, maize, wheat, rice, fig, spices and cocoa during pre- and post-harvest conditions (Mahato et al. 2019). Besides these food products, commercial products like peanut-butter, cooking oil and cosmetics have also been reported to be contaminated by aflatoxins. Foods contaminated with lower concentrations of aflatoxin are toxic to both human and animals.

Abass et al. (2017) concluded that some food processed from cassava were considered safe for human consumption due to the low amount of mycotoxin observed in the samples tested such as *garri, fufu* (1.2 µg/kg of aflatoxin B1) and cassava flour (2.9 µg/kg of aflatoxin G1) compared to the European Union safe toxic levels of 5 µg/kg and the report of Ediage et al. (2011) in the republic of Benin. Akinmusire et al. (2019) detected AFB1 with 83% incidence in feed samples ranging from 0.5–760 µg/kg compared to aflatoxin B2, aflatoxin G1, aflatoxin G2 and aflatoxin M1 found at a lower concentration in food and feed ingredient tested. High rate of fungal-producing aflatoxins in feed samples has been reported across different countries such as Cameroon (Njobeh et al. 2012), India, Nigeria (Kehinde et al. 2014), and South Africa (Kotinagu et al. 2015).

Across SSA especially in Nigeria, where chilli peppers are fundamental ingredients in many traditional dishes; Ezekiel et al. (2019a) examined 70 samples of chilli pepper from Kano, Nasarrawa and Oyo and reported over 25% of aflatoxin concentration in the samples which was higher than the maximum consumption limit. About 69% of samples collected were contaminated with 8.9 ppb aflatoxin. The report indicated that Oyo state had the highest of contamination of 15.2 ppb, of which 45% of samples contaminated have a concentration of AFB1 which was significantly higher than the EU maximum limit. This suggests that dry pepper intake is a potential spot for chronic aflatoxin exposure among Nigerians giving the fact that it is often added to nearly all forms of meals. This too could hinder marketability of the country’s agricultural produce as agro-commodity like pepper contaminated with mycotoxin could be turned down as produce that exceeds tolerable limit are restricted importation into nations where aflatoxins are strictly regulated.

Different species of the genera *Aspergillus, Penicillium, Cladosporium, Alternaria, Fusarium* and *Acremonium* were isolated in maize grains samples from Kano and Plateau Opadokun and Ikeorah (1983), as well as from Aba, Abakaliki, Afikpo, Okigwe and Owerri – all in southeast Nigeria by Aja‐Nwachukwu and Emejuaiwe (1994). These fungi species are implicated as aflatoxicogenic in common foodstuff. Pepple et al. (2016) observed the presence of total aflatoxin in healthy (3.7 ± 0.02 iµ/kg) and infected (3.8 ± 0.01 iµ/kg) shelved fruits of *Dialium guineense* which was below the 4.0 ± 0.15 µg/kg standards set by National Agency for Food and Drug Administration (NAFDAC); the agency responsible for regulations of safe food and food materials in Nigeria .

According to Ruby et al. 2013) cited in Olajuyigbe et al. (2014), feeding aflatoxin contaminated feed to fish caused a significant reduction in growth of different fish and shrimp species.

Obani et al. (2019) compared infected rate of aflatoxigen fungi-producing aflatoxins in six different melon popularly known as *egusi* in Nigeria and showed that aflatoxin-B (6.9– 109.5 µg/kg) and aflatoxin-G (0.9– 35.8 µg/kg) were detected in all types of melon tested for aflatoxin. Various food condiments such as melon are prone to aflatoxin contamination at levels exceeding the permissible limits indicated by Food and Drug Administration (FDA). This is an indication that all types of melon are significant source of aflatoxin contamination and hence, are potential sources of exposure to the harmful effects of aflatoxin when consumed.

In the southern part of Nigeria, where melon is highly cultivated, market women are prompted to dry and stored the seeds of melon in a containers prior marketing. Dried seeds of melon are further process by grinding in to powder which are later stored in diverse form of containers. Stored ground melons are could be contaminated by mycotoxin due to their exposure to environmental factors that favours growth of fungi. Bread a major breakfast product in many parts of Africa, especially in Nigeria was confirm across various states in Nigeria to contain aflatoxin beyond the safe limit by Ojochenemi et al. (2019). Onyedum et al. (2020), reported 100% occurrence of aflatoxin contamination in millet, sorghum and yam flour at a range (4.80–248.20 μg/kg) beyond the safe limit of 4 μg/kg set by Commission of the European Communities (CEC). The myriads of results obtained from different parts of Nigeria pointed to the fact that intake of aflatoxin contaminated feed is a major challenge in human health and the national economy.

### Ochratoxin A contamination of food and feed in Nigeria

Ochratoxin A (OTA) is a mycotoxin commonly associated with many foods, feeds and beverages produce in tropical and temperate regions. Literature on OTA contamination in food and feeds in Nigeria are very scanty. Although, Makun et al. (2013) reveals a high prevalent of OTA in maize, a common ingredient of many food and feed. Higher concentration of OTA beyond the EU regulatory limit of 5 ug/kg in raw cereal grain was recorded in Niger State, The reports on awareness and prevalence of mycotoxin contamination in selected Nigerian fermented foods, which comprised maize gruel (*ogi*), sorghum gruel (*ogi-baba*), melon seed (*ogiri*), locust bean (*iru*) and African oil bean seed (*ugba*) showed that OTA concentration in *ogi-baba*, (6 µg/kg) *iru* (6 µg/kg) and *ugba* (9 µg/kg) was above the recommended limit set by CEC for food stuff (Adekoya et al. 2017a). However, Oyelami et al. (1996) reported absence of OTA in *ogi*. This could be as a result of non-participation of fungi-producing OTA in *Ogi* during the fermentation process. The study of Makun et al. (2013) showed that virtually all 107 out of 109 Nigerian foods and feed commodities tested were contaminated by the nephrotoxic OTA at concentration above 5 mg/kg regarded as unsafe by the EU (CEC (Commission of the European Communities) 2006). Bankole and Adebanjo (2003) and Dongo et al. (2008) reported detection OTA in cocoa beans and its products. The results of Dongo et al. (2008) confirmed the present of OTA at concentration ranging from 1 to 277.5 µg/kg and very few reports of OTA incidence in other crops are available in Nigeria.

### Zearalenone (ZEN) contamination of food and feed

According to Kpodo et al. (1996), zearalenone (ZEN) is largely produced by some *Fusarium* spp. in temperate region at very low temperature between 10°C and 15°C, whereas temperatures from 27°C to 40°C commonly persist yearly in Nigeria. This explains the reason why most food samples tested positive for ZEN at a lesser level compare to the maximum limit recommended in about 16 countries. However, Logrieco et al. (2018) reported that in Africa, the contamination of **z**earalenone mycotoxin maybe be a concern. Zearalenone has been reported to be a major mycotoxin in contaminated agricultural products in Nigeria (Adejumo et al. 2007). Chilaka et al. (2018a) confirmed the present of ZEN in *garri, lafun* and *yam* flour at an incidence rate of 17% (17 µg/kg), 6% (16 µg/kg), and 9% (19 µg/kg), respectively. In a study by Oyeka et al. (2019), the ZEN contamination of maize in Anambra State was higher than the tolerable limit set by the EC 2006. Cashew nuts purchase from different location in Nigeria by Adetunji et al. (2019) was ascertain to have ZEN concentration of 788 µg/kg higher than the maximum limit of 75 µg/kg proposed by the EC no 1126/2007. This could be a threat to human as well as animal health because ZEN produces oestrogen effect leading to hyperstrogen.

### Fumonisins contamination of food and feed

Fumonisins appear to be the second most significant mycotoxin in Nigeria and other tropical countries. The consequence of consuming food contaminated with fumonisin especially FB1 has played a role in human oesophageal cancer. Akinmusire et al. (2019), detected 23 fungal mycotoxin including emerging ones from poultry and feed ingredient collected from different part of Nigeria. Fumonisins was observed as the most frequent mycotoxin in the feed with an incidence rate of 97% fumonisin B1 that ranges from 37–3760 µg/kg. Researches have also shown a relatively high significant concentration of fumonisins in poultry feeds in Nigeria (Ezekiel et al. 2012) and other African countries such as Cameroon (Abia et al. 2013b) and South Africa (Njobeh et al. 2012). The high rate of contamination could be as a result of climatic factors, handling, storage practices for raw materials, and formulation mix utilised when compounding the feeds. Several studies have also revealed the prevalence of fumonisin in Nigeria food commodities (Adejumo et al. 2007; Chilaka et al. 2016; Adetunji et al. 2019).

In 2015, the International Agency for Research on Cancer (IARC) reported the possible occurrence and association of mycotoxins such as fumonisins which causes stunting in young children. Unlike the developed countries; there are no strict regulatory standard in SSA, which protects young infant with lower body mass, high metabolic rate, immature organ function and detoxification mechanism from secondary fungal metabolite which are detrimental to their health. (Peraica et al. 2014)

### Deoxynivalenone (DON) contamination of food and feed

Chilaka et al. (2018b) examined safety of traditional beers and spices contaminated with *Fusarium* spp. from Nigeria market. They reported the present of DON to be 65% incidence and contamination ranged from 65–185 µg/L which was lower compared to 93% recorded in brewed maize beer (Abia et al. 2013b) and no DON incidence in Malawian maize beer (Matumba et al. 2014). This could be linked to stability of toxin during the brewing processing or lower amount of samples taken into consideration by the researcher.

In another study conducted by Akoma et al. (2019), stored maize in Kogi State, Nigeria were found to be contaminated with DON at a range of 1.34– 9.25 µg/kg out of which 65% were above the Joint Expert Committee for Food Additives (JECFA) provisional tolerable maximum daily intake (PTMDI) of 1 µg/kg for human consumption. In a mini survey carried out by Egbontan et al. (2017) on mycotoxin in locally grown wheat, DON was 100% present among other toxins with concentration ranging from 119 to 2560 μg/kg equivalent of 36% more than the European Union tolerable limit of 750 µg/kg in wheat.

### T-2 contamination of food and feed

The prevalence of Fusarium mycotoxins in cassava and yam were determined using multi-mycotoxin liquid chromatography-tandem mass spectrometry (LC-MS/MS) by Chilaka et al. (2018a). The level of T2 in *garri* was reported to have a lower concentration than the EU recommendation level of 50 µg/kg. *Garri* is consumed generally by all Nigerians both rich and the poor but more common among the low income earners which implies that majority are predisposed to the toxic effect of T-2.

## Problems associated with mycotoxin research in Nigeria

Research of mycotoxin in Nigeria started in the year 1961 instigated by a group of researcher from Tropical Research Institute in UK with knowledge of aflatoxin relating to the world and the significance of its spread. However, lot of challenges have hindered application of its findings to control its damaging effect on food safety, human and animal health as well as national economy. Some of the constraints against effective adoption of mycotoxin research findings or outputs include lack of equipment or limited kits, inadequate facilities for safe mycotoxin research, misused of reagents, lack of maintenance of laboratory facilities, poor funding and inadequate skills,

### Lack of equipment and limited available kits

Detection of mycotoxin has been limited to use of thin layer chromatography (TLC) because of poor funding to acquire up-to-date equipment that can provide better precision and accurate information. The use of out-dated equipment makes any result coming from this part of the world suspicious and its reliability questionable. This poses serious challenge to researchers embarking on mycotoxins research. Recently, new techniques such as HPLC, ELIZA LC-MS/MS have proven to present reliable results ranging from smaller to larger samples. Fewer universities in Nigeria have HPLC while there is no facility for LC-MS/MS in nearly all public universities. Although, researchers sometimes collaborate with colleagues in some private universities that have acquired these equipment but at some cost. Consequent upon the huge cost of procuring these equipment, researchers whose work centres on mycotoxin study spend a lot of funds to get their samples analysed through a third party arrangement. Many who work on detection and quantification of mycotoxin in some food and feed samples send such to institutions or laboratories with modern facilities for analyses usually, at a enormous cost. Besides, in some cases, samples are sent overseas countries with known or proven best standard laboratory procedures whose accuracy, precision and reliability meet global best standards.

### Inadequate facilities for safe mycotoxin research

One of the major factors that hindered the success of mycotoxin research in Nigeria is the poor or inadequate awareness information on toxicity of mycotoxin. Many are weary and unwilling to venture into mycotoxin research because of its associated hazard especially where there lack of basic personal protective equipment. Exposure of personnel to mycotoxin research using crude safety gears and equipment may lead to health complications hence researchers working on food safety tends to avoid mycotoxin related trials because of the high risk involved.

### Misused of reagent

Reagents should be readily available and access by researchers. Mycotoxin reagents are usually imported generally in developing countries most especially, Nigeria. One of the challenges with acquiring reagents and mycotoxin standards from foreign companies is the scepticism and trepidation of its use for biological weapons instead as against research purposes (Atanda et al. 2013). In spite of the myriads of these hiccups, scientists from these lowly developed economies have remain resolute in their approach to ensuring food safety.

### Lack of maintenance of laboratory facilities

To obtained reliable results that are reproducible, laboratories equipment must be properly cleaned. All equipment used for mycotoxin in Africa countries such as Nigeria are imported for a few that can afford it and highly trained personnel may not be available for proper maintenance and regular routine checked.

### Poor funding and inadequate skills

Government in many developing countries have paid little or insignificant attention to research funding in the area of food safety as it relates to mycotoxin contamination. Although, groups of some researchers under the aegis of Mycotoxicological Society of Nigeria (MSN) has a mandate to research into mycotoxin related studies but the society lacks governmental support needed to effectively deliver their mandate. In Nigeria there is currently no Government agency or Department that specifically provides funds for or promotes mycotoxin research. Many have collaborated with the International Institute of Tropical Agriculture (IITA) and other well-funded institutes to carry on with researches and create awareness program necessary to disseminate and sensitise the public on the effect of mycotoxins (Bankole and Adebanjo 2003).

In Nigeria, there is shortage of trained personnel to embark on seminars, conferences and workshops that discuss mycotoxin contaminations and its implication on food safety. Skills are gained only through cheap training workshops and conferences organised by MSN in collaboration with Mycotoxin kits producers. However, the needed supports from government agencies such as the Standards Organization of Nigeria (SON) and National Agency for Food and Drug Administration and Control (NAFDAC) to carry out national research on mycotoxins is not in existence of which training can only be done through special arrangement.

Non-governmental bodies like International Institute of Tropical Agriculture (IITA), Ibadan has been so helpful by providing supports in form of capacity building as well as providing bench work service though at exorbitant cost. Without government intervention defraying funding cost and providing a forum or program to sensitised the populace on mycotoxin contamination mycotoxin. The unsafe levels of mycotoxin contamination of food and feeds will continue unabated and reduce the market value of the food stuff and as well casting aspersion on unmarketability of agricultural produce. Sadly, while a number of developed countries and some sub-sectors in the African regions have achieved notable progress in implementing food safety sustainability and improvement strategies, many including Nigeria, Uganda and others are still struggling to tailor their traditional food control systems to current food safety (Obinna 2015).

## Regulations of mycotoxin in Nigeria

Mycotoxins contaminate food, feed, or raw materials used in their production and cause diseases and many disorders in humans and livestock. Because of their great variety of toxic effects and their extreme heat resistance, the presence of mycotoxin in food and feed is considered a high risk to human and animal health. In order to ensure food quality and health of animals and consumers, many countries have regulations guiding mycotoxin concentrations in difference produce. European legislation has set maximum contents of some mycotoxins in different matrices (Arroyo-Manzanares et al. 2014). Contamination of food and feed by fungi-producing mycotoxin has remained a great challenge to global food safety of public health and economic significance. Different countries have set a limit of contamination to different food and feed to mitigate the health challenges traceable to mycotoxins contamination. This is ensure safety of food thereby preventing development of mycotoxins induced health issues such as carcinogenicity, hepatotoxicity, and teratogenicity, weakened immune systems, body poisoning through respiration, and mutilation of DNA structure (Razzaghi-Abyaneh et al. 2014; Akoma et al. 2019). Several limits as shown in Table 2 have been set by regulatory agencies such as the European Union. However, in Nigeria, the only existing mycotoxin regulation is for aflatoxin and fumonisins (Bandyopadhyay et al. 2007). Nigeria have adopted the European commission and Codex Alimentarius Standards mycotoxin regulations which are used primarily for export commodities (Atanda et al. 2013; Adejumo and Adejoro 2014). The Codex Alimentarius Standards for aflatoxin limit is 0.5 ug/kg for milk and its products and beans and nuts as stated in Table 2. Currently there is no tolerable limit of trichothecene in Nigeria.

**Table 2.** Regulation of mycotoxin used in comparison with safe to eat food and feeds

**Mycotoxins**	**Commodities**	**Maximum acceptable limit**	**Legislative body**
Aflatoxin	milk and its products, beans	0.5μg/kg	Codex Alimentarius Standard
	nuts: peanuts &almonds,	4 to 5μg/kg	
	Cereals.	10μg/kg 20μg/kg	
	Melon	2μg/kg AFB1	EU (Adopted by Nigeria)
		4 μg/kg AFT	
	All cereals and all products derived from cereals	2.0 μg/kg (AFB1) 4.0 μg/kg (AFT)	European union (EU)
Fumonisins	Unprocessed cereals	<1000 μg/kg	(CAST), 2003) accepted by Nigeria.
	Unprocessed maize	4000 μg/kg	EU
	Maize intended for direct human consumption	1000 μg/kg	
Ochratoxin A	raw cereals	0.5μg/kg in 0.12μg/kg weekly	EU/ Codex Alimentarius
	wine and juice and	2μg/kg	
	Unprocessed cereals wheat, barley, rye	5.0 μg/kg	
	Spice	20 μg/kg	
DON	processed cereal based foods and baby foods for infants and young children	200μg/kg	(Commission of the European Communities, 2006)
	Cereal grains (wheat, maize and barley) for processing.	2000 μg/kg	Codex Alimentarius
	Unprocessed wheat, oats and maize	1750μg/kg	EU
Zearalenone	Unprocessed cereals and Maize intended for direct human consumption	100 μg/kg	EU
	Unprocessed maize	350 μg/kg	
	Cereals intended for direct human consumption	75 μg/kg	

Nevertheless, regulations have been set to prevent and control the occurrence of mycotoxin in industrially processed foods, and those meant for export in many developed countries with no concern for locally processed goods where no effort is made towards the control of toxigenic fungi in food contamination especially in developing countries where there is little or no regulations (Mu et al. 2017). Regulations set throughout the world by the European Union and other organisations adopted by developing nations including Nigeria, do not consider the multiple combined effects of mycotoxins. Equally, there is no extant regulation that could be adapted or enforced to reduce the rate of contamination caused in victuals materials by these mycotoxins (Ferrão et al. 2017).

Generally in the developing countries and some part of developed countries, exposure to mycotoxins especially excessive high fumonisins by populations that consumed maize and its products cannot be addressed by regulatory standards (Shephard et al. 2019). Multifaceted approach should be applied in the regulating the limit of food and feed contaminated by mycotoxins-producing fungi. This is because enforcement of regulation of maximum limit is not practical in subsistence farming system where the food produced are meant for household consumption and little are traded within communities. Therefore maximum regulatory limit should be set to favour rejection of produce for both subsistence and commercial farmers.

Unacceptable trade practices where mouldy food materials are mixed with wholesome or high-quality products to maximise profit also persists due to non-enforcement of regulatory limits. This is a common practice among produce entrepreneurs or middle-men that buy and sell agro-commodities in local markets. Aside from this ignorance of the existence of mycotoxins is another major challenge among the rural small produce holders. Many produce including maize, cereals, coffee, spices, peanuts, pistachio nuts and other nuts meant for exportation with high risk of mycotoxin contamination, spread across many continents. However, prevalence rate differ from one part of the world to the other due to environmental factors, storage techniques and methods of handling. More recently, between 2015 and 2016, the European Union rejected 67 foods including: sesame seeds, melon seeds, peanut chips, live snails, ginger, prawns, dried meat and fish, mushrooms, palm oil, bitter leaf, cowpea, crayfish due to failure to meet regulatory standards on aesthetics and bacterial contamination, appearance of mould on the samples, and failure of the food products to meet their pesticide requirements (Ogunfuwa 2017).

The study by Somorin et al. (2016) concerning AFs, OTA, and citrinin co-occurrence in egusi melon seed from Nigeria is one of the examples which explains the basis for increasing border rejection of melon seed consignments from Nigeria to EU as indicated in European Rapid Alert System for Food and Feed (RASFF). This led to the enactment of legislation which mandates that 50% of consignments of *egusi* and their derived products from Nigeria be checked before being allowed entry into the EU (Kleter et al. 2009; Marvin et al. 2009). So, many nations and agencies have come up with its own standardisation of maximum accepted limit of mycotoxin contamination of food and feeds. Stating a maximum acceptable limit of contamination needs to be harmonised in both food and feeds concerning the safety of human and animal health. According to (Mu et al. 2017) this can only be done by having a common agency that can assess and generalised the maximum limit from various regulating body to adopt a common limit.

## Possible strategies for the control of mycotoxin in food and feed in Nigeria

A number of strategies for preventing mycotoxins have been proposed in different areas of the world including African countries but the awareness for implementation is very low (Mu et al. 2017). In developing countries like Nigeria and the rest of Africa, food safety is an important issue in public health and mycotoxin contaminated food and feed is a huge safety risk. A lot of control measures have been discussed in literatures (Bankole and Adebanjo 2003; Atanda et al. 2013; Chilaka et al. 2017; Sokefun et al. 2018; Adebiyi et al. 2019). Possible strategies for the control strategies of mycotoxins in Nigeria involves: Good agricultural practices, physical separations, use of media, proper storage conditions, detoxification/degradation and food fermentation.

### Good agricultural practice

Good agricultural practices (GAP) is a collection of principles laid down for farmers to apply for pre-production and post-production that will result in safe and healthy agricultural and non-agricultural product (Atanda et al. 2013). It represents a primary line of defence against mycotoxins contamination in food and feed. This should be follow by good manufacturing practice during handling, storage processing and distribution of food and feed for human and animal consumptions. GAP such as early harvesting and proper drying have been shown to have contributed to an effective control of mycotoxin contamination that have resulted to a reduction in aflatoxin infestation (Hell et al., 2010). In Nigeria, the best possible way to reduced contamination is from good agricultural practices. Nigeria has not kept pace with the adoption of good agricultural practices, which included food safety regulations. It is very important to reach out to farmers in every state for awareness on GAP. Alleviation of mycotoxin contamination will be successful if all good agricultural practices are in place by farmer in Nigeria.

### Physical separation

Sorting and proper cleaning are considered to be the first step of physical elimination of mycotoxins. They might be regarded as superior methods since they pose no risk of producing toxic degradable products (Chilaka et al. 2017). Several other methods such as washing, dehulling, hand picking of visible mouldy have been reported to be an efficient physical decontamination methods of different mycotoxin types. Researches have shown that fumonisin are not uniformly distributed in grains that are stored together and according to the study of Afolabi et al. (2006), visible sorting of grain was suggested as a simple technique that can be used to reduce exposure to the fumonisins. Good quality grain can as well be contaminated with fumonisins on a less significant level (Desjardins et al. 1998) compared to poor quality one that are sorted. Sorting would have been a good method of segregating good and bad quality grains only if the bad quality ones are discarded and not being diverted to other uses such as animal feed or mixed with good quality and ground to powder.

### Proper storage conditions

Storage is a critical stage where infection and mycotoxin accumulation occur. Storage conditions play a significant role in controlling mycotoxins contamination since they affect the overall growth of fungi. Storage under controlled conditions, such as packaging practices, temperature control, ventilation, and appropriate air humidity, reduce the growth of fungi and the accumulation of mycotoxins. Care must be taken to store grains that are wholesome and apparently healthy. Pre-storage treatment or handling should take care of certain basic issues. Before storage, rapid drying of farm produces is highly recommended for tackling mycotoxins problems since all factors leading to contaminations are linked to safe moisture content. Studies have shown that the level of aflatoxin increases with the time of storage in hot and humid countries such as Nigeria and are more at risk due to the combination of heat and dampness that favours the growth of *Aspergillus* and *Fusarium* common producers of mycotoxin (Villers 2014). Proper storage mechanisms with adequate ventilation are necessary to prevent fungal-producing mycotoxin (Atukwase et al. 2012). Several preventive measures have been suggested through research but not fully implemented by the public.

### Creation of aggressive awareness on prevalence and danger of mycotoxins

Lack of awareness of the dangers posed by fungi that produce mycotoxins is a major factor responsible for its high incidence in Nigeria (Atanda et al. 2013). The use of media to create the much needed awareness is a good strategy for control, reduction and elimination of mycotoxin contamination of foods and feeds. Studies have shown that the unawareness level of mycotoxin contamination is slightly correlated with the level of education in some part of Nigeria. Majority of farmers, food handlers and food processors are illiterate with virtually no knowledge of the implications of mycotoxin contaminations. Awareness program could be performed through government bodies, private organisations, and national media networks using newspapers and magazines as well as preparation of seminar and workshop that are used as avenue and bridge of information exchange and dissemination between researchers and citizens (Tola and Kebede 2016). Creating awareness on the effect of implementation of GAP, Good management Practices (GMP), and Hazard Analysis Critical Control Points (HACCP) in the control of mycotoxin contamination in Nigeria food system will be ideal to some extent in reducing the risk of mycotoxins exposure in both the rural and urban communities.

### Detoxification/degradation and food fermentation

Biological elimination has been reported to be one of the best methods in controlling and preventing mycotoxins in food and feeds. This has been proven with the use of Aflasafe, a biocontrol product containing microbial strains (Fungal strains) that controls aflatogenic fungi that produces aflatoxins in maize and groundnut. Aflasafe product is currently available in few African countries such as Burkina Faso, Ghana, Kenya, Nigeria, Senegal and The Gambia, with indications of imminent availability in other African countries (Adebiyi et al. 2019).

Another universal biological food processing method is fermentation. In Nigeria and other SSA countries, fermentation is considered as one of the most technologically and appropriate methods for food processing due to its affordability and suitability for the production of staple foods in rural and urban regions (Adebisi et al. 2019). It improves food quality and provides spectacular properties for consumers. It can serve as an alternative technique to reduce mycotoxins compared to costly and impractical techniques. The establishment of a strong and an efficient food control and regulation system is the key component of ensuring food safety. There is therefore need to safeguard the quality and safety of food both for domestic consumption and for exportation.

## Conclusions

This review examined the safety of mycotoxin contamination of food and feed in Nigeria and equally established the need to improve awareness of stakeholders involve in agro-commodities enterprises on the harmful effect of fungi that produce mycotoxins and associated health risks. Considering the safety of consumers, strategies towards mitigation mycotoxin contamination should be prioritised. In developing countries such as Ghana, Burkina Faso, Nigeria and others faced with high poverty and illiteracy rate, it may be an up-hill task to eradicate incidence of mycotoxin contamination in agricultural produce.

However, adopting the use of good agricultural practices, good manufacturing practices, good storage practices, proper farming techniques and planting resistance varieties will go a long way in minimising the concentration of mycotoxin in agricultural commodities both for domestic use and international trade. Properly designed, and constant mycoflora and mycotoxin surveys; monitoring programmes, provision of adequate diagnostic facilities can also reduce fungal and mycotoxins in our foods. Considering the safety of consumers, strategies towards mycotoxin reduction to acceptable standards should be a prioritised. It is high time Nigeria enforced the legislation against mycotoxins contaminated food and feeds to ensure they are safe from mycotoxin contamination and its attendant effects of the consumers.

## References

[cit0001] Abass AB, Awoyale W, Sulyok M, Alamu EO. 2017. Occurrence of regulated mycotoxins and other microbial metabolites in dried cassava products from nigeria. Toxins. 9(7). doi:10.3390/toxins9070207. PMC5535154 28661436

[cit0002] Abdallah MF, Audenaert K, Lust L, Landschoot S, Bekaert B, Haesaert G, De Boevre M, De Saeger S. 2020. Risk characterization and quantification of mycotoxins and their producing fungi in sugarcane juice: a neglected problem in a widely-consumed traditional beverage. Food Control. 108(April 2019). doi:10.1016/j.foodcont.2019.106811.

[cit0003] Abia WA, Simo GN, Warth B, Sulyok M, Krska R, Tchana A, Moundipa PF. 2013a. Determination of multiple mycotoxins levels in poultry feeds from cameroon. Japanese Journal of Veterinary Research. 61(Issue SUPPL.):S61–S63. 23631150

[cit0004] Abia WA, Warth B, Sulyok M, Krska R, Tchana AN, Njobeh PB. 2013b. Determination of multi-mycotoxin occurrence in cereals, nuts and their products in Cameroon by liquid chromatography tandem mass spectrometry (LCMS/MS). Food Control. 21(2):438e453.

[cit0005] Adebiyi JA, Kayitesi E, Adebo OA, Changwa R, Njobeh PB. 2019. Food fermentation and mycotoxin detoxification: an African perspective. Food Control. 106(May):106731. doi:10.1016/j.foodcont.2019.106731.

[cit0006] Adejumo TO, Adejoro DO. 2014. Incidence of aflatoxins, fumonisins, trichothecenes and ochratoxins in Nigerian foods and possible intervention strategies. Food Science and Quality Management. 31(September):127–147.

[cit0007] Adejumo TO, Hettwer U, Karlovsky P. 2007. Survey of maize from south-western Nigeria for zearalenone, α- and b-zearalenols, fumonisin β1 and enniatins produced by fusarium species. Food Addit Contam. 24(9):993–1000. doi:10.1080/02652030701317285. 17691013

[cit0008] Adekoya I, Njobeh P, Obadina A, Chilaka C, Okoth S, De Boevre M, De Saeger S. 2017a. Awareness and prevalence of mycotoxin contamination in selected nigerian fermented foods. Toxins. 9(11):1–16. doi:10.3390/toxins9110363. PMC5705978 29117141

[cit0009] Adekoya I, Njobeh P, Obadina A, Chilaka C, Okoth S, De Boevre M, De Saeger S. 2017b. Awareness and prevalence of mycotoxin contamination in selected nigerian fermented foods. Toxins. 9(11):1–16. doi:10.3390/toxins9110363. PMC5705978 29117141

[cit0010] Adekoya I, Obadina A, Adaku CC, De Boevre M, Okoth S, De Saeger S, Njobeh P. 2018. Mycobiota and co-occurrence of mycotoxins in South African maize-based opaque beer. Int J Food Microbiol. 270(2017):22–30. doi:10.1016/j.ijfoodmicro.2018.02.001. 29453120

[cit0011] Adetunji MC, Aroyeun SO, Osho MB, Sulyok M, Krska R, Mwanza M. 2019. Fungal metabolite and mycotoxins profile of cashew nut from selected locations in two African countries. Food Additives and Contaminants - Part A Chemistry, Analysis, Control, Exposure and Risk Assessment. 36(12):1847–1859. doi:10.1080/19440049.2019.1662951. 31535946

[cit0012] Adjovi YCS, B Jg G, Bailly S, Bailly JD, Tadrist S, Puel O, Oswald IP, Sanni A. 2015. Occurrence of mycotoxins in cassava (manihot esculenta crantz) and its products. International Journal of Food Safety, Nutrition and Public Health. 5(3/4):217. doi:10.1504/ijfsnph.2015.070157.

[cit0013] Afolabi CG, Bandyopadhyay R, Leslie JF, Ekpo EJA. 2006. Effect of sorting on incidence and occurrence of fumonisins and fusarium verticillioides on maize from Nigeria. J Food Prot. 69(8):2019–2023. doi:10.4315/0362-028X-69.8.2019. 16924936

[cit0014] Afolabi CG, Ekpo EJA, Bandyopadhyay R. 2013. Maize contamination by zearalenone and T-2 toxin and human exposure in Nigeria. Mycotoxins. 63(2):143–149.

[cit0015] Afolabi CG, Ezekiel CN, Ogunbiyi AE, Oluwadairo OJ, Sulyok M, Krska R. 2020. Fungi and mycotoxins in cowpea (*vigna unguiculata* L) on Nigerian markets. Food Additives and Contaminants: Part B Surveillance. 13(1):52–58. doi:10.1080/19393210.2019.1690590. 31739763

[cit0016] Aja‐Nwachukwu J, Emejuaiwe SO. 1994. Aflatoxin‐producing fungi associated with Nigerian maize. Environmental Toxicology and Water Quality. 9(1):17–23. doi:10.1002/tox.2530090104.

[cit0017] Akinmusire OO, El-Yuguda AD, Musa JA, Oyedele OA, Sulyok M, Somorin YM, Ezekiel CN, Krska R. 2019. Mycotoxins in poultry feed and feed ingredients in Nigeria. Mycotoxin Res. 35(2):149–155. doi:10.1007/s12550-018-0337-y. 30484071 PMC6478637

[cit0018] Akoma ON, Ezeh CC, Chukwudozie KI, Iwuchukwu CC, Apeh DO. 2019. Fungal and Mycotoxin contamination of Stored Maize in Kogi, Northcentral Nigeria: an implication for public health. European Journal of Nutrition & Food Safety. doi:10.9734/ejnfs/2019/v9i330061.

[cit0019] Alshannaq A, Yu JH. 2017. Occurrence, toxicity, and analysis of major mycotoxins in food. Int J Environ Res Public Health. 14(6). doi:10.3390/ijerph14060632. PMC5486318 28608841

[cit0020] Arroyo-Manzanares N, Huertas-Pérez JF, García-Campaña AM, Gámiz-Gracia L. 2014. Mycotoxin analysis: new proposals for sample treatment. Advances in Chemistry. 2014:1–12. doi:10.1155/2014/547506.

[cit0021] Atanda O, Makun HA, Ogara IM, Edema M, Idahor KO, Eshiett ME, Oluwabamiwo BO. 2013. Fungal and Mycotoxin contamination of Nigerian foods and feeds. Mycotoxin and Food Safety in Developing Countries, April. doi:10.5772/55664.

[cit0022] Atukwase A, Kaaya AN, Muyanja C. 2012. Dynamics of fusarium and fumonisins in maize during storage - A case of the traditional storage structures commonly used in Uganda. Food Control. (1). doi:10.1016/j.foodcont.2012.01.016.

[cit0023] Apeh DO, Ochai DO, Adejumo A, Muhammad HL, Saidu AN, Atehnkeng J, Adeyemi RH, Mailafiya SC, Makun HA. 2016. Mycotoxicological Concerns with Sorghum, Millet and Sesame in Northern Nigeria. J Anal Bioanal Tech. 7:336. doi:10.4172/2155-9872.1000336.

[cit0024] Bandyopadhyay R, Kumar M, Leslie J. 2007. Relative severity of aflatoxin contamination of cereal crops in West Africa. Food Addit Contam. (10). doi:10.1080/02652030701553251. 17886182

[cit0025] Bankole SA, Adebanjo A. 2003. Mycotoxins in food in West Africa: current situation and possibilities of controlling it. African Journal of Biotechnology. 2(9):254–274. doi:10.5897/AJB2003.000-1053.

[cit0026] CAST (Council for Agricultural Science and Technology). 2003. *Mycotoxins: risks in plant, animal, and human systems. Task Force Report No. 139*.

[cit0027] CEC (Commission of the European Communities). 2006. Commission Regulation (EC) no. 1881/2006 of 19 December 2006 setting maximum levels for certain contaminants in foodstuffs. Official Journal of the European Union. L364:5e24.

[cit0028] Chilaka CA, De Boevre M, Atanda OO, De Saeger S. 2016. Occurrence of fusarium mycotoxins in cereal crops and processed products (Ogi) from Nigeria. Toxins. 8(11):1–18. doi:10.3390/toxins8110342. PMC5127138 27869703

[cit0029] Chilaka CA, De Boevre M, Atanda OO, De Saeger S. 2017. The status of fusarium mycotoxins in sub-Saharan Africa: a review of emerging trends and post-harvest mitigation strategies towards food control. Toxins. (1). doi:10.3390/toxins9010019. PMC5308251 28067768

[cit0030] Chilaka CA, De Boevre M, Atanda OO, De Saeger S. 2018a. Prevalence of fusarium mycotoxins in cassava and yam products from some selected Nigerian markets. Food Control. 84:226–231. doi:10.1016/j.foodcont.2017.08.005.

[cit0031] Chilaka CA, De Boevre M, Atanda OO, De Saeger S. 2018b. Quantification of fusarium mycotoxins in Nigerian traditional beers and spices using a multi-mycotoxin LC-MS/MS method. Food Control. 87:203–210. doi:10.1016/j.foodcont.2017.12.028.

[cit0032] Chilaka CA, De Boevre M, Atanda OO, De Saeger S. 2018c. Stability of fumonisin B1, deoxynivalenol, zearalenone, and T-2 toxin during processing of traditional Nigerian beer and spices. Mycotoxin Res. 34(4):229–239. doi:10.1007/s12550-018-0318-1. 29725912

[cit0033] Chilaka CA, De Boevre M, Atanda OO, De Saeger S. 2019. Fate of fusarium mycotoxins during processing of Nigerian traditional infant foods (ogi and soybean powder). Food Research International. 116:408–418. doi:10.1016/j.foodres.2018.08.055. 30716963

[cit0034] Cinar A, Onbaşı E. 2019. Mycotoxins: the hidden danger in foods. Mycotoxins and Food Safety [Working Title]. doi:10.5772/intechopen.89001.

[cit0035] Darwish WS, Ikenaka Y, Nakayama SMM, Ishizuka M. 2014. An overview on Mycotoxin contamination of foods in Africa. J Vet Med Sci. 76(6):789–797. doi:10.1292/jvms.13-0563. 24572628 PMC4108760

[cit0036] Desjardins AE, Plattner RD, Lu M, Claflin LE. 1998. Distribution of fumonisins in maize ears infected with strains of fusarium moniliforme that differ in fumonisin production. Plant Disease. doi:10.1094/PDIS.1998.82.8.953. 30856929

[cit0037] Dikhoba PM, Mongalo NI, Elgorashi EE, Makhafola TJ. 2019. Antifungal and anti-mycotoxigenic activity of selected South African medicinal plants species. Heliyon. 5(10):e02668. doi:10.1016/j.heliyon.2019.e02668. 31692684 PMC6806395

[cit0038] Dongo L, Bandyopadhyay R, Kumar M, Ojiambo PS. 2008. Occurence of Ochratoxin A in Nigerian for Nigeria ready for sale cocoa beans. Agricultural Journal. 3(1):4–9.

[cit0039] Ediage EN, Di Mavungu JD, Monbaliu S, Van Peteghem C, De Saeger S. 2011. A validated multianalyte LC-MS/MS method for quantification of 25 mycotoxins in cassava flour, peanut cake and maize samples. J Agric Food Chem. 59(10):5173–5180. doi:10.1021/jf2009364. 21495720

[cit0040] Egbontan AO, Afolabi CG, Kehinde IA, Enikuomehin OA, Ezekiel CN, Sulyok M, Warth B, Krska R. 2017. A mini-survey of moulds and mycotoxins in locally grown and imported wheat grains in Nigeria. Mycotoxin Res. 33(1):59–64. doi:10.1007/s12550-016-0264-8. 27905064

[cit0041] Egbuta M, Wanza M, Dutton M. 2015. Evaluation of five major Mycotoxins Co-contaminating two cereal grains from Nigeria. International Journal of Biochemistry Research & Review. 6(4):160–169. doi:10.9734/ijbcrr/2015/15306.

[cit0042] Esan AO, Fapohunda SO, Ezekiel CN, Sulyok M, Krska R. 2020. Distribution of fungi and their toxic metabolites in melon and sesame seeds marketed in two major producing states in Nigeria. Mycotoxin Res. (4). doi:10.1007/s12550-020-00400-0. PMC7536151 32666399

[cit0043] Ezekiel CN, Ortega-Beltran A, Oyedeji EO, Atehnkeng J, Kössler P, Tairu F, Hoeschle-Zeledon I, Karlovsky P, Cotty PJ, Bandyopadhyay R. 2019a. Aflatoxin in chili peppers in Nigeria: extent of contamination and control using atoxigenic aspergillus flavus genotypes as biocontrol agents. Toxins. (7). doi:10.3390/toxins11070429. PMC6669588 31336571

[cit0044] Ezekiel CN, Sulyok M, Ogara IM, Abia WA, Warth B, Šarkanj B, Turner PC, Krska R. 2019b. Mycotoxins in uncooked and plate-ready household food from rural northern Nigeria. Food and Chemical Toxicology. doi:10.1016/j.fct.2019.04.002. 30965105

[cit0045] Ezekiel CN, Sulyok M, Warth B, Odebode AC, Krska R. 2012. Natural occurrence of mycotoxins in peanut cake from Nigeria. Food Control. 27(2):338–342. doi:10.1016/j.foodcont.2012.04.010.

[cit0046] Ferrão J, Bell V, Fernandes TH. 2017. Mycotoxins, food safety and security in Sub-Saharan Africa. SM Journal of Foood and Nutritional Disorders. 3(2):1–9. https://smjournals.com/food-nutritional-disorders/download.php?file=fulltext/smjfnd-v3-1021.pdf.

[cit0047] Gruber-Dorninger C, Jenkins T, Schatzmayr G. 2019. Global mycotoxin occurrence in feed: a ten-year survey. Toxins. 11(7). doi:10.3390/toxins11070375. PMC6669473 31252650

[cit0048] Gruber-Dorninger C, Novak B, Nagl V, Berthiller F. 2017. Emerging Mycotoxins: beyond traditionally determined food contaminants. J Agric Food Chem. 65(33):7052–7070. doi:10.1021/acs.jafc.6b03413. 27599910

[cit0049] Hell K, Mutegi C, Fandohan P. 2010. Aflatoxin control and prevention strategies in maize for Sub-Saharan Africa. Julius-Kühn-Archiv, 425: 535. doi:10.5073/jka.2010.425.388.

[cit0050] Ikeagwulonu RC, Onyenekwe CC, Oshim IO, Olise NA, Odeyemi O, Ojidei CK. 2020. Investigation of the levels of total Aflatoxin in herbal traditional medicines from selected vendors dealers in South-Eastern Nigeria. Journal of Advances in Medical and Pharmaceutical Sciences. doi:10.9734/jamps/2020/v22i130152.

[cit0051] JPF D, Macdonald AMC. 1997. Mycotoxins. Animal Feed Science and Technology. 69(1–3):155–166. doi:10.1016/S0377-8401(97)81630-6.

[cit0052] Kebede H, Liu X, Jin J, Xing F. 2020. Current status of major mycotoxins contamination in food and feed in Africa. Food Control. 110:106975. doi:10.1016/j.foodcont.2019.106975.

[cit0053] Kehinde MT, Oluwafemi F, Itoandon EE, Orji FA, Ajayi OI. 2014. Fungal Profile and Aflatoxin Contamination in poultry feeds sold in Abeokuta, Ogun State, Nigeria. Nigerian Food Journal. 32(1):73–79. doi:10.1016/s0189-7241(15)30098-9.

[cit0054] Kleter GA, Prandini A, Filippi L, Marvin HJP. 2009. Identification of potentially emerging food safety issues by analysis of reports published by the European community’s rapid alert system for food and feed (RASFF) during a four-year period. Food and Chemical Toxicology. (5). doi:10.1016/j.fct.2007.12.022. 18255210

[cit0055] Kotinagu K, Mohanamba T, Rathna Kumari L. 2015. Assessment of aflatoxin B1 in livestock feed and feed ingredients by high-performance thin layer chromatography. Veterinary World. 8(12):1396–1399. doi:10.14202/vetworld.2015.1396-1399. 27047050 PMC4774816

[cit0056] Kpodo K, Sørensen AK, Jakobsen M. 1996. The occurrence of mycotoxins in fermented maize products. Food Chem. 56(2):147–153. doi:10.1016/0308-8146(95)00155-7.

[cit0057] Ladeira C, Frazzoli C, Orisakwe OE. 2017. Engaging one health for non-communicable diseases in Africa: perspective for Mycotoxins. Frontiers in Public Health. 5(October). doi:10.3389/fpubh.2017.00266. PMC5650707 29085817

[cit0058] Lippolis V, Irurhe O, Porricelli ACR, Cortese M, Schena R, Imafidon T, Oluwadun A, Pascale M. 2017. Natural co-occurrence of aflatoxins and ochratoxin A in ginger (zingiber officinale) from Nigeria. Food Control. 73:1061–1067. doi:10.1016/j.foodcont.2016.10.026.

[cit0059] Logrieco AF, Miller JD, Eskola M, Krska R, Ayalew A, Bandyopadhyay R, Battilani P, Bhatnagar D, Chulze S, De Saeger S, et al. 2018. The mycotox charter: increasing awareness of, and concerted action for, minimizing mycotoxin exposure worldwide. Toxins. (4). doi:10.3390/toxins10040149. PMC5923315 29617309

[cit0060] Mahato DK, Lee KE, Kamle M, Devi S, Dewangan KN, Kumar P, Kang SG. 2019. Aflatoxins in food and feed: an overview on prevalence, detection and control strategies. Front Microbiol. 10(October):1–10. doi:10.3389/fmicb.2019.02266. 31636616 PMC6787635

[cit0061] Makun HA, Adeniran AL, Mailafiya SC, Ayanda IS, Mudashiru AT, Ojukwu UJ, Jagaba AS, Usman Z, Salihu DA. 2013. Natural occurrence of ochratoxin A in some marketed Nigerian foods. Food Control. 31(2):566–571. doi:10.1016/j.foodcont.2012.09.043.

[cit0062] Makun HA, Dutton MF, Njobeh PB, Mwanza M, Kabiru AY. 2011. Natural multi-occurrence of mycotoxins in rice from Niger State, Nigeria. Mycotoxin Res. 27(2). doi:10.1007/s12550-010-0080-5. PMC3150825 21836766

[cit0063] Marvin HJP, Kleter GA, Prandini A, Dekkers S, Bolton DJ. 2009. Early identification systems for emerging foodborne hazards. Food and Chemical Toxicology. (5). doi:10.1016/j.fct.2007.12.021. 18272277

[cit0064] Mata AT, Ferreira JP, Oliveira BR, Batoréu MC, Barreto Crespo MT, Pereira VJ, Bronze MR. 2015. Bottled water: analysis of mycotoxins by LC-MS/MS. Food Chem. 176:455–464. doi:10.1016/j.foodchem.2014.12.088. 25624256

[cit0065] Matumba L, Van Poucke C, Biswick T, Monjerezi M, Mwatseteza J, De Saeger S. 2014. A limited survey of mycotoxins in traditional maize based opaque beers in Malawi. Food Control. 36(1):253–256. doi:10.1016/j.foodcont.2013.08.032.

[cit0066] Mu U, Cg O, Muritala A. 2017. An Overview of Mycotoxin contamination of foods and feeds. Journal of Biochemical and Microbial. 1(1):1–11.

[cit0067] Muhammad HK, Apeh DO, Muhammad HL, Olorunmowaju YB, Ifeji E, Makun HA. 2019. Mycoflora of maize in Niger State, Nigeria. Advanced Research in Life Sciences. 3(1):40–45. doi:10.2478/arls-2019-0009.

[cit0068] Neji PA, Vincent TO, Anozie RC. 2018. Assessment of the levels of Mycotoxins in varieties of cereals (Oryza Sativa, Zea Mays, Pennisetum Glaucum and Triticum Aestivum) obtained from calabar markets, Cross River State, Nigeria. International Journal of Scientific and Research Publications (IJSRP). 8(4):393–398. doi:10.29322/ijsrp.8.4.2018.p7655.

[cit0069] Njobeh PB, Dutton MF, Åberg AT, Haggblom P. 2012. Estimation of multi-mycotoxin contamination in South African compound feeds. Toxins. 4(10):836–848. doi:10.3390/toxins4100836. 23162700 PMC3496991

[cit0070] Obani FT, Atehnkeng J, Ikotun B, Bandyopadhyay R. 2019. Natural occurrence of Aflatoxin in different egusi types found in Nigeria. Issue 1 Ser II. 12:15–20. doi:10.9790/2380-1201021520.

[cit0071] Obinna C 2015. Food safety: how safe is food in Nigeria?. *Vanguard*. https://www.vanguardngr.com/2015/04/food-safety-how-safe-is-food-in-nigeria/

[cit0072] Ogunfuwa I 2017. EU rejects 67 Nigerian foods in two years. *Punch New Paper*. http://www.punchng.com/eu-rejects-67-nigerian-foods-two-years

[cit0073] Ogungbemile AO, Etaware PM, Odebode AC. 2020. Aflatoxin detection and quantification in stored cowpea seeds in Ibadan, Nigeria. Journal of Biotechnology and Biomedicine. 03(02):10–18. doi:10.26502/jbb.2642-91280022.

[cit0074] Ojochenemi AD, Oku UP, Anthony MH. 2019. Mutual occurrence and dietary exposure to total Aflatoxin and fumonisins in bread: a major breakfast bakery product in Nigeria. Advanced Research in Life Sciences. 3(1):33–39. doi:10.2478/arls-2019-0008.

[cit0075] Olajuyigbe OO, Akande GR, Ezekiel CN, Ezekiel MO. 2014. Aflatoxigenic moulds and aflatoxin contamination of retailed fishery products in Lagos markets. Mycotoxicology Society of Nigeria Mycotoxicology. 1:57–63.

[cit0076] Olufunmilayo GO, Oyefolu AB. 2010. Natural occurrence of aflatoxin residues in fresh and sun-dried meat in Nigeria. Pan Afr Med J. 7(August):14. doi:10.4314/pamj.v7i1.69124. 21918701 PMC3172626

[cit0077] Omeiza GK, Kabir J, Kwaga JKP, Kwanashie CN, Mwanza M, Ngoma L. 2018. A risk assessment study of the occurrence and distribution of aflatoxigenic aspergillus flavus and aflatoxin B1 in dairy cattle feeds in a central northern state, Nigeria. Toxicology Reports. 5(August):846–856. doi:10.1016/j.toxrep.2018.08.011. 30151345 PMC6107895

[cit0078] Onyedum SC, Adefolalu FS, Muhammad HL, Apeh DO, Agada MS, Imienwanrin MR, Makun HA. 2020. Occurrence of major mycotoxins and their dietary exposure in North-Central Nigeria staples. Scientific African. 7:e00188. doi:10.1016/j.sciaf.2019.e00188.

[cit0079] Opadokun JS, Ikeorah J. 1983. Moisture and aflatoxin contents of market grain samples in Kano and Plateau States of Nigeria.

[cit0080] Oyeka CA, Amasiani RN, Ekwealor CC. 2019. Mycotoxins contamination of maize in Anambra State, Nigeria. Food Additives and Contaminants: Part B Surveillance. 12(4):280–288. doi:10.1080/19393210.2019.1661528.

[cit0081] Oyelami OA, Maxwell SM, Adeoba E. 1996. Aflatoxins and ochratoxin A in the weaning food of Nigerian children. Ann Trop Paediatr. (2). doi:10.1080/02724936.1996.11747816. 8790677

[cit0082] Pankaj SK, Shi H, Keener KM. 2018. A review of novel physical and chemical decontamination technologies for aflatoxin in food. Trends in Food Science and Technology. 71(May 2019):73–83. doi:10.1016/j.tifs.2017.11.007.

[cit0083] Pepple GA, Chukunda FA, Ukoima HN. 2016. Detection of fungi and Aflatoxin contamination in shelved fruits of Dialium guineense Wild, Rivers State, Nigeria. Asian Journal of Plant Science and Research. 6(1):1–5.

[cit0084] Peraica M, Richter D, Rašić D. 2014. Mycotoxicoses in children. Arh Hig Rada Toksikol. 65(4):347–363. doi:10.2478/10004-1254-65-2014-2557. 25720023

[cit0085] Razzaghi-Abyaneh M, Chang PK, Shams-Ghahfarokhi M, Rai M. 2014. Global health issues of aflatoxins in food and agriculture: challenges and opportunities. Front Microbiol. doi:10.3389/fmicb.2014.00420. PMC4129441 25161651

[cit0086] Ruby DS, Masood A, Fatmi A. 2013. Effect of Aflatoxin contaminated feed on growth and survival of fish labeo rohita (hamilton). Current World Environment Journal. 8(3):479–482. doi:10.12944/cwe.8.3.19.

[cit0087] Shephard GS, Burger HM, Rheeder JP, Alberts JF, Gelderblom WCA. 2019. The effectiveness of regulatory maximum levels for fumonisin mycotoxins in commercial and subsistence maize crops in South Africa. Food Control. 97(August 2018):77–80. doi:10.1016/j.foodcont.2018.10.004.

[cit0088] Sokefun E, Ayepola OO, Olasehinde GI. 2018. Mycotoxins: food production and exportation in Nigeria. IOP Conf Ser: Earth Environ Sci. 210(1). doi:10.1088/1755-1315/210/1/012018.

[cit0089] Somorin Y, Akinyemi A, Bertuzzi T, Pietri A. 2016. Co-occurrence of aflatoxins, ochratoxin A and citrinin in “egusi” melon (colocynthis citrullus L.) seeds consumed in Ireland and the United Kingdom. Food Additives and Contaminants: Part B Surveillance. (3). doi:10.1080/19393210.2016.1183051. 27134068

[cit0090] Tola M, Kebede B. 2016. Occurrence, importance and control of mycotoxins: a review. Cogent Food & Agriculture. 2(1):1–12. doi:10.1080/23311932.2016.1191103.

[cit0091] Van Der Fels-Klerx HJ, Liu C, Battilani P. 2016. Modelling climate change impacts on mycotoxin contamination. World Mycotoxin Journal. 9(5):717–726. doi:10.3920/WMJ2016.2066.

[cit0092] Villers P. 2014. Aflatoxins and safe storage. Front Microbiol. doi:10.3389/fmicb.2014.00158. PMC3989759 24782846

